# Wearable Sensors System for an Improved Analysis of Freezing of Gait in Parkinson’s Disease Using Electromyography and Inertial Signals

**DOI:** 10.3390/s19040948

**Published:** 2019-02-23

**Authors:** Ivan Mazzetta, Alessandro Zampogna, Antonio Suppa, Alessandro Gumiero, Marco Pessione, Fernanda Irrera

**Affiliations:** 1 Department of Information Engineering, Electronics and Telecommunication, Sapienza University of Rome, 00184 Rome, Italy; fernanda.irrera@uniroma1.it; 2 Department of Human Neurosciences, Sapienza University of Rome, 00185 Rome, Italy; alessandro.zampogna@gmail.com (A.Z.); antonio.suppa@uniroma1.it (A.S.); 3 IRCSS NEUROMED Institute, 86077 Pozzilli IS, Italy; 4 STMicroelectronics, 20864 Agrate Brianza MI , Italy; alessandro.gumiero@st.com (A.G.); marco.pessione@st.com (M.P.)

**Keywords:** wearable sensors, sensor fusion, inertial signal, surface electromyography, gait analysis, Parkinson’s disease, telemedicine

## Abstract

We propose a wearable sensor system for automatic, continuous and ubiquitous analysis of Freezing of Gait (FOG), in patients affected by Parkinson’s disease. FOG is an unpredictable gait disorder with different clinical manifestations, as the trembling and the shuffling-like phenotypes, whose underlying pathophysiology is not fully understood yet. Typical trembling-like subtype features are lack of postural adaptation and abrupt trunk inclination, which in general can increase the fall probability. The targets of this work are detecting the FOG episodes, distinguishing the phenotype and analyzing the muscle activity during and outside FOG, toward a deeper insight in the disorder pathophysiology and the assessment of the fall risk associated to the FOG subtype. To this aim, gyroscopes and surface electromyography integrated in wearable devices sense simultaneously movements and action potentials of antagonist leg muscles. Dedicated algorithms allow the timely detection of the FOG episode and, for the first time, the automatic distinction of the FOG phenotypes, which can enable associating a fall risk to the subtype. Thanks to the possibility of detecting muscles contractions and stretching exactly during FOG, a deeper insight into the pathophysiological underpinnings of the different phenotypes can be achieved, which is an innovative approach with respect to the state of art.

## 1. Introduction

Freezing of Gait (FOG) is a paroxysmal gait disorder affecting patients with Parkinson’s disease (PD) and representing a leading cause of falls and poor quality of life [1,2,3]. FOG consists in the sudden inability to generate steps and forward feet progression, subjectively perceived as feet “glued to the ground” [4].

The frequency and the duration of FOG episodes are heterogeneous and depend on numerous factors including the stage of disease, the pharmacological state of therapy, the context of evaluation and emotional stimuli [4].

The knowledge of FOG pathophysiology, currently enigmatic, is the prerequisite for a targeted therapy that can help patients in preventing catastrophic falls as consequence of involuntary motor blocks during gait. According to leg motion observed during FOG episodes, at least two main phenotypes of FOG can be clinically identified [5,6], one of which, the so-called *trembling in place* FOG is associated with the inability to lift the foot from the ground and to an alternating tremor of the legs (knee trembling) with no effective forward feet progression. In the *trembling in place* FOG, the rapid variation of body acceleration due to the toe stuck to the ground and knee trembling is accompanied by the lack of postural adaptation and the abrupt inclination of trunk angle, which in general can cause forward falls during gait on the linear path and lateral falls during turning [6,7,8,9].

Another FOG subtype is the *shuffling forward* FOG, which is a less disabling form of FOG consisting of short, shuffling steps and a minimal forward feet displacement.

In addition, there is the akinetic FOG, a very uncommon form not showing any limbs or trunk movement, thus recognizable only by the patient.

Recent experimental evidence shows that the aforementioned FOG subtypes may have different underlying neurophysiological underpinnings [10].

Based on the wide heterogeneity of FOG clinical manifestations and pharmacological responses [5,7,11], recent experimental evidence highlights the need to perform appropriate FOG subtype classification to potentially uncover different underlying pathophysiological mechanisms and then therapeutic implications [7].

The transition from the stance to the swing phase of gait, impaired during FOG episodes, involves a specific pattern of muscles activation with a distal to proximal trend [12].

Accordingly, the study of temporal characteristics of the high-frequency lower-limb oscillation during FOG (knee trembling) repeats a distal to proximal pattern of muscles activation [13].

Previous studies shows a “toe-off” deficit in patients with PD and FOG, analyzing the activation of specific distal leg muscles with electromyography (EMG). Some authors indicated a reduced activation of the gastrocnemius muscle (GC) [14,15], an early activation [16] or even a hyper-activation of the tibialis anterior muscle (TA) [14], as well as an increased co-activation of GC and TA muscles [17] during gait in patients with PD and FOG. However, some of these studies consider gait only outside FOG episodes [14,16] often in a fixed laboratory environment, such as a treadmill [14]. Furthermore, some authors only considered the activity of one muscle without evaluating the synergic activity of the two antagonist muscles [15]. Finally, previous studies show both paroxysmal and continuous gait disturbances [18,19].

In this frame, clarifying the activation pattern of GC and TA muscles during and outside FOG episodes would definitely allow new insights into the pathophysiology of the disorder and alternative therapeutic strategies.

Much work has been done in the last decade in the field of telemedicine with respect to the home assistance of patients affected by chronic diseases and significant effort has been devoted in particular to wearable sensors for the detection of motion symptoms [20,21,22,23,24,25]. In the specific context of the gait analysis and FOG detection in PD, inertial measurement units (IMU) are mainly used [24,25,26,27,28,29,30,31,32,33,34,35,36,37,38,39,40,41,42,43,44,45,46,47]. In very few cases, different signals are considered. For example, in [48], electrocardiogram and skin conductance data are used to analyze FOG and try to predict FOG episodes by identifying specific pattern changes in both electrocardiogram and skin conductance traces during pre-FOG, FOG and post-FOG situations respect to regular gait. In [49], dynamical machine-learning algorithms are designed to track the presence and severity of tremor and dyskinesia with 1-s resolution by analyzing signals collected from PD patients wearing hybrid sensors with both 3-D accelerometer and surface EMG.

In this paper, we propose a wearable sensors system for automatic, continuous and ubiquitous monitoring of specific gait features in free living-like conditions, for real time and remote assistance of patients. It uses contemporarily gyroscope (gyro) and surface EMG (sEMG) for detecting FOG episodes. Fusion of the gyro and sEMG signals distinguishes the *trembling in place* and the *shuffling forward* FOG subtypes, thus allowing the off-line quantification of these phenomena and the associated risk of falls. This experimental approach would help to assess possible differences in response to treatments in the two distinct subtypes of FOG. To our knowledge, no previous study has performed an objective distinction between *trembling in place* and *shuffling forward* FOG subtypes by means of wearable devices. Finally, this system also helps to analyze the activation pattern of the limb antagonist muscles during and outside FOG episodes, recognizing the status of stretching and contraction.

Our wearable system has some innovative aspects with respect to the others presented in the literature. First, using distinct and independent signals of different nature, namely an angular velocity and the sEMG, we enlarge the extent and increase the reliability of the information regarding the gait disorder respect to using only data from an IMU.

Furthermore, this is the first time that *trembling in place* and *shuffling forward* phenotypes of FOG are distinguished by using a wearable device. Such distinction is relevant for a better comprehension of the underlying pathophysiology, for predicting associated risk of falls and finally for clarifying response to treatments, in the two distinct subtypes of FOG, in patients with PD. Once the relationship between FOG subtype and fall risk is assessed by our system, the possibility to monitor incidence and phenotype of the FOG episodes of a patient along the day would define an individual fall risk and significantly improve the quality of life.

Finally, to our knowledge, we are presenting the first attempt to perform a systematic analysis of the activation pattern of antagonist muscles (GC and TA) during and outside FOG episodes in a free living-like environment, as opposed to previous studies [16,17,18] using fixed laboratory tools.

Results of this innovative study can represent a pathway toward a deep insight into the pathophysiology of the FOG and in the neurophysiological underpinnings of the different phenotypes and open new therapeutic strategies, such as proprioceptive manipulation to improve patient outcomes.

**Figure 1:**
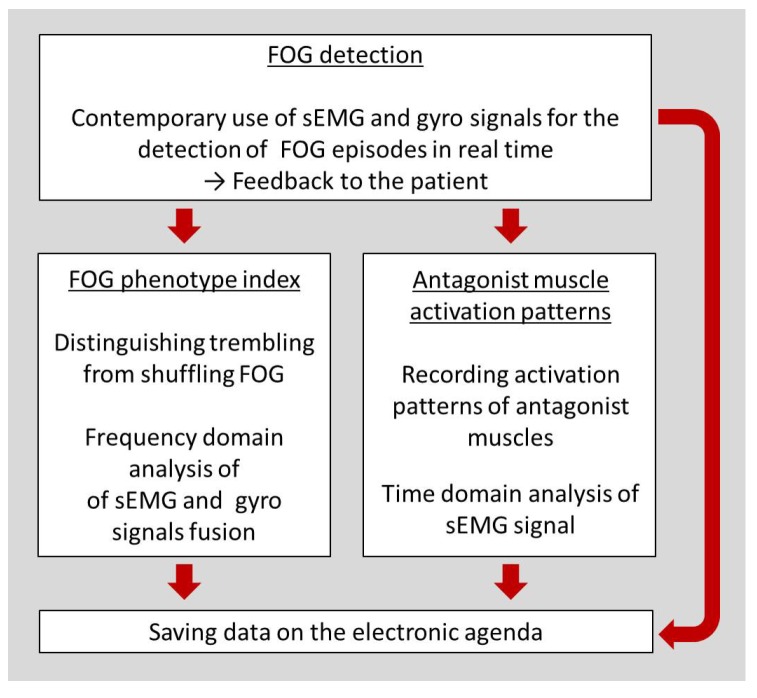
Sketch of the paper flow.

The paper flow is depicted in Figure 1, where the three main work topics are outlined. The algorithm for the real-time FOG detection using gyro and sEMG is introduced in Section 3. The results from tests on PD patients are discussed in the same section.

The real-time detection of FOG would allow timely alerting of patients or caregivers to implement recovery strategies. In Section 4, the frequency-domain processing of the sEMG and gyro signals fusion for distinguishing the *trembling in place* from the *shuffling* FOG is discussed and a FOG phenotype index is proposed. In Section 5, we present results regarding the type (contraction or stretching) and the intensity of the antagonist muscles activity during and outside FOG episodes obtained after sEMG signal processing.

All data can be stored for the realization of an electronic agenda to be off-line analyzed in a clinical setting by doctors. In the next section, the hard device and components of the proposed system are described.

## 2. Materials

**Figure 2:**
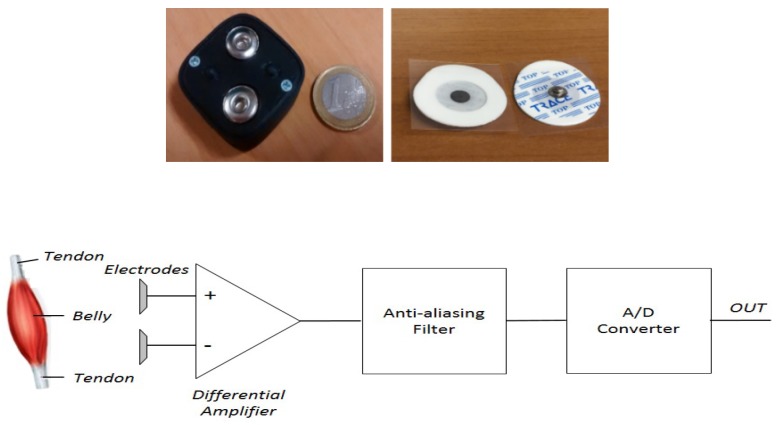
Back-side of the Bio2Bit Move (**top left**); patch electrodes (**top right**); and bio-potential system block diagram used for sEMG acquisition (**bottom**).

The hard device is the prototype Bio2Bit Move developed by STMicroelectronics (Agrate Brianza, Italy), shown in Figure 2. It includes: an ultra-low power bio-potential acquisition system with one differential channel for sEMG acquisition (ST HM121), an inertial measurement unit (IMU LSM6DS3H) with a ±16 g 3D accelerometer and a ±2000 dps 3D gyroscope, a 32 bit computational unit ARM^®^ Cortex^®^-M4, a microSD, a low-energy Bluetooth 4.0, a 592 mWh battery, and a micro-USB connector. The device dimensions are 30 mm × 30 mm × 15 mm, and the weight is 10 g including the battery. The IMU collects data with a sample frequency up to 6.6 kHz. It integrates an analog anti-aliasing low pass filter, with a selectable cut off frequency up to 400 Hz, which can be disabled in the mode high-performance.

The gyro exhibits an angular rate accuracy down to 4.375 mdps/LSB, an angular rate typical zero-rate level of ±10 dps and a root mean square (RMS) noise in low power mode of 120 mdps. The accelerometer exhibits a linear acceleration sensitivity down to 0.061 mg/LSB, a zero-*g* level offset accuracy of ±40 mg and a RMS noise in low power mode of up to 4.4 mg. Two clips are integrated into the device package, as shown in Figure 2 (top left). The adhesive electrode patches pictured in Figure 2 (top right) and buttoned in the clips record the sEMG signal on the skin surface. The distance between the clips is 20 mm and positioning of the patches can be done by non-specialized users in a domestic environment. Preliminary feasibility tests investigating the correlation between the system performance and the device positioning have been previously performed [50]. Further unpublished data are available on request. The sEMG signal detected by electrodes is amplified because of the attenuation due to the skin and the subcutaneous tissue. The block diagram of the bio-potential system (NM121) for sEMG signal acquisition is displayed in Figure 2 (bottom). The maximum amplification provided by HM121 is 128, whereas the common mode rejection ratio (CMRR) (at 50 Hz) and the signal to noise ratio (SNR) (with a signal of 10 Hz, 10 mVpp, gain 64) are, respectively, 65 dB and 59 dB. The integrated bandpass filter features bandwidth 0.5–300 Hz, to avoid aliasing effects and to eliminate the DC component. The integrated ADC features 14 bit resolution and ±0.6 V reference voltage. The measured overall noise is 38 μV, in agreement with requirements for medical applications (<50 μV). The device collects data in real time with a sampling frequency up to 4 kHz.

In this work, devices were positioned on the GC and/or on the TA. They recorded contemporarily the inertial state by means of gyro and the muscular activity by means of sEMG. Regarding the sEMG, the caregivers or in some cases the patients themselves, all previously trained by expert doctors, placed the adhesive electrode patches over the GC and TA muscles in line with standardized international criteria [51,52]. In more detail, sEMG from GC muscle was recorded by placing electrodes at about 1/3 of the line between the head of the fibula and the heel, on the lateral mass of the calf during plantar-flexion of the foot, with the knee extended. Differently, sEMG from TA muscle was recorded by placing electrodes at about 1/3 of the line between the tip of the fibula and the tip of the medial malleolus, laterally to the tibial crest, during dorsiflexion of the foot.

There is a relatively high tolerance on the electrodes positioning on GC and TA since the signal is recorded on long and wide muscle bundles. In the case of an error with respect to the exact placing point, the problem would only be an attenuation of the signal. However, we demonstrated that the system performance did not depend on the operator who positioned the device. In conclusion, we are confident that patients and caregivers can be trained to place correctly the electrodes with the aid of explanatory images, thus allowing the use of the device in a domestic environment.

**Figure 3:**
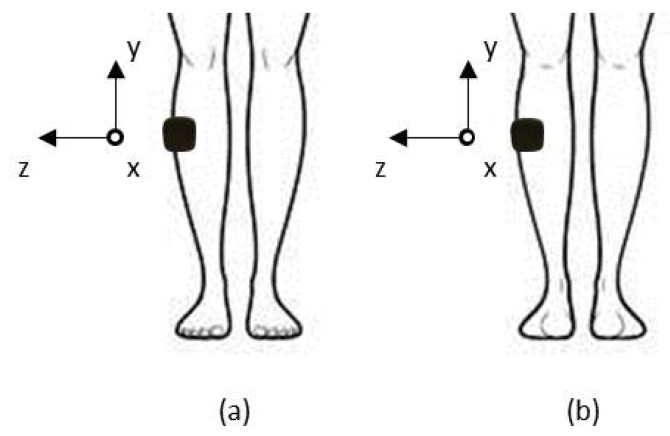
Sketch of the device positioning on: (**a**) the tibialis anterior; and (**b**) the gastrocnemius of the right leg. The axis orientation is reported.

The position on the GC and on the TA is sketched, respectively, in Figure 3a,b, where the gyro axis orientation is also indicated. Referring to Figure 3, we considered the angular velocity around the z-axis, which lies in the frontal plane and well represents the human motion during linear gait for devices positioned on both TA and GC.

## 3. Real Time FOG Detection and Results

### 3.1. Methods

Tests were performed on seven subjects showing a very wide variety of disease features, in order to be as general as possible. Patients gave written informed consent and the experimental procedures were approved by the institutional review board of Sapienza University in accordance with the Declaration of Helsinki. Patients were males aged 65–79, with a disease duration from 5 to 13 years, values of the FOG questionnaire from 8 to 23, a Hoen and Yahr scale from 2 to 3 [53] and a Modified Disorder Society-Unified Parkinson’s disease Rating Scale (MDS-UPDRS) [54] pars III in the range of 27 to 51 and in the range of 18 to 36 in OFF and ON cases, respectively. All patients repeated eight times the test: four times in OFF condition (after drug withdrawal for at least 12 h) and four times in ON condition (1 h after the administration of usual dopaminergic treatment). Patients performed a 7-m TUG test consisting of: getting up from a chair, walking for 7 m, turning (both right-side and left-side), walking back for 7 m, and sitting down. The TUG test was carried out in an ecological setting reproducing a domestic environment with possible obstacles eliciting FOG, such as the passage thorough a narrow corridor (approximatively 1.5 m wide), the approach to an open door and the presence of furniture. Indeed, the test ambient was more similar to a domestic environment than a biomedical laboratory where conventional sEMG measurement is usually performed with movements limited in space and constricted by wires. In this sense, hereafter we address to the test condition as performed in a free living-like environment.

All tests were filmed and the electronic device recordings synchronized with the videos. Two doctors, experts in the movement disorders field, separately and independently inspected the videos and stated the exact starting and ending time of each FOG episode and the specific FOG phenotype. The doctors’ statements were assumed as our reference. Evaluation of the system performance in FOG recognition, and calculation of false positive and false negative, were based on the comparison with the reference. The total recording time was 57 min (for the 56 tests), during which doctors recognized 99 FOG episodes, mainly in the linear gait and mainly of the *trembling in place* phenotype. Tests lasted from 39 s to 348 s, with an average duration of 61 s. The FOG occurrence per test was from 0 s to 16 s. In particular, three subjects experienced FOG of both phenotypes and one of these by himself collected 40 episodes in total, both in ON and in OFF condition (20 *trembling in place* and 20 *shuffling forward*). The other four subjects experienced only of the *trembling in place* subtype. No one had only *shuffling* FOG episodes. Although the number of studied subjects is limited, the amount of FOG episodes is quite high. Therefore, this work can be considered statistically meaningful even if the set of individual features in which the FOG manifested is related to only seven subjects.

**Figure 4:**
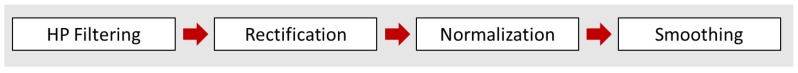
Stages of a conventional preprocessing of the raw sEMG signal.

Following the literature [50,55,56], we performed the conventional preprocessing resumed in Figure 4. In particular, the sEMG raw signal was high-pass (HP) filtered at 3 Hz to remove both the hum noise and the motion artifacts. It was rectified by calculating its absolute value. It was normalized to its maximum value. Finally, it was smoothed using low-pass (LP) filtering at 10 Hz to obtain the envelope. Hereafter, we address to the so-processed raw signal simply as sEMG signal.

**Figure 5:**
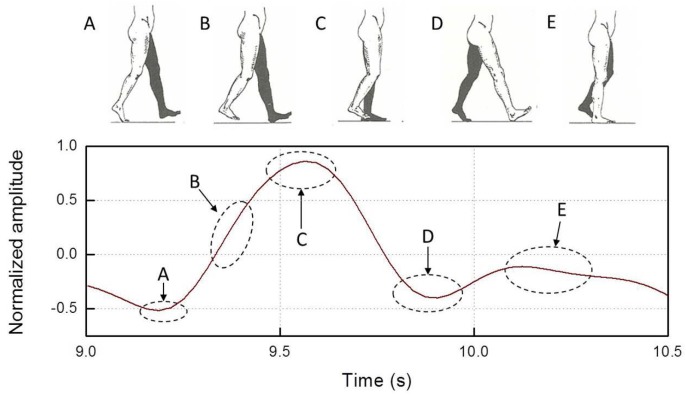
An example of the normalized angular velocity around the z-axis recorded on the TA in correspondence of the distinct phases of a single step: (**A**) toe-off; (**B**) initial swing; (**C**) mid-swing; (**D**) heel strike; and (**E**) mid-stance.

For the sake of clarity, in Figure 5, we report a typical gyro trace recorded during a single regular step, with the correspondence to the distinct step phases [57] indicated. The gyro trace was low-pass filtered at 3 Hz to highlight the regular gait frequency content (0–3 Hz) [28].

**Figure 6:**
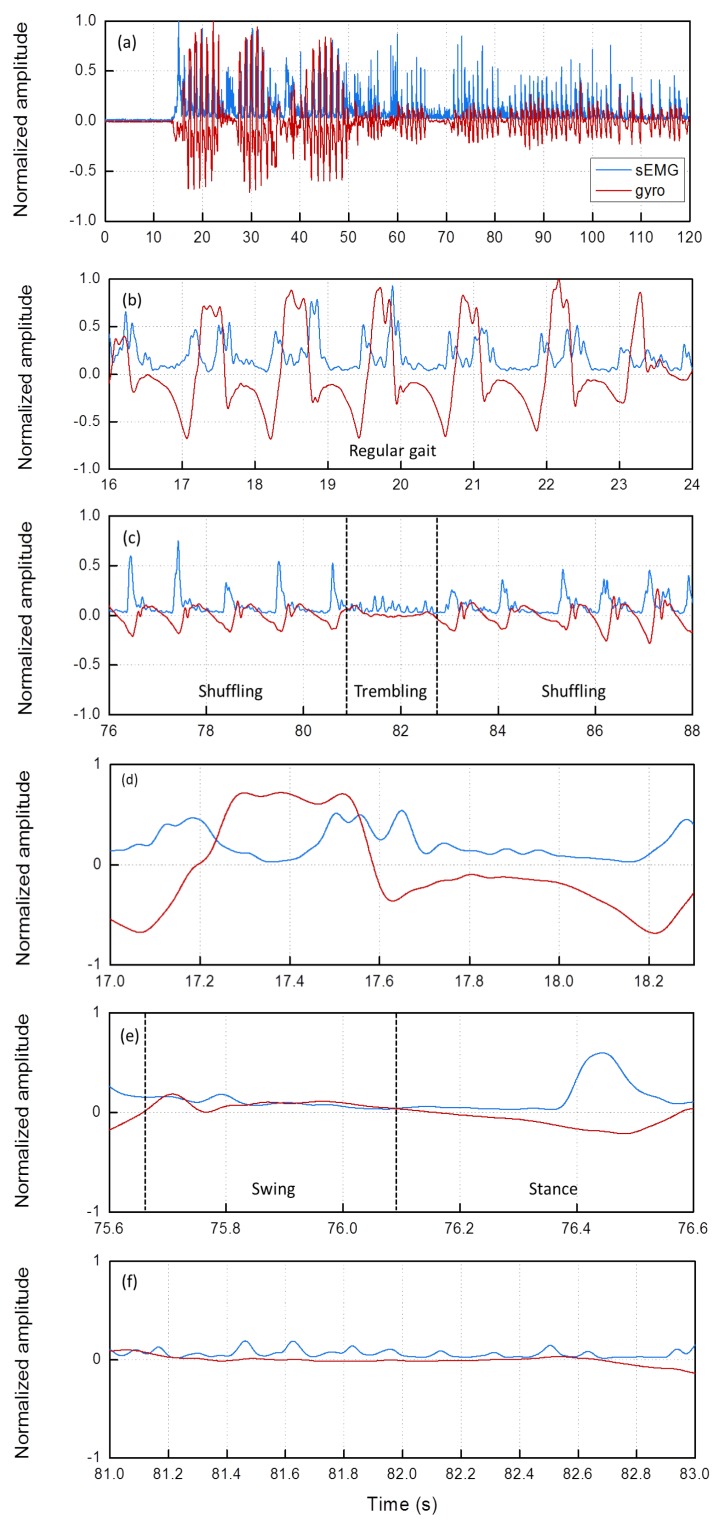
(**a**) An example of the sEMG (blue) and gyro (red) traces recorded on the TA; (**b**) zoom of the trace in a regular gait interval; (**c**) zoom of the trace in the FOG interval; (**d**) zoom of a single regular step; (**e**) zoom of a single shuffled step; and (**f**) zoom of step attempts during *trembling* FOG.

In Figure 6a, an example of the gyro and the (preprocessed) sEMG traces recorded on the TA of a patient during a test is shown. Both traces were normalized with respect to their absolute maximum for enabling comparison among different subjects exhibiting traces with different amplitude. During time *t* = 0–13 s, the patient was sitting (rest). Then, he got up from the chair and started walking regularly with a turning (rightward) around *t* = 25 s and went back to sitting on the chair (*t* = 38 s). Then, again, around *t* = 40 s, he got up from the chair and started walking regularly, with a turning (leftward) around *t* = 50 s.

After that the patient experienced a long FOG with both the *shuffling* and *trembling in place* phenotypes. The first FOG interval (50–65 s) refers to a FOG episode during turning. Then, for a few seconds, the patient stood up in a rest condition. After that, for *t* > 69 s, he experienced *shuffling* and *trembling in place* FOG episodes during gait in the linear path.

Zooms of specific portions of the traces are displayed in Figure 6b (during regular gait) and Figure 6c (during FOG). In the zoom of Figure 6b, we clearly see that, during the swing phase of each regular step, the sEMG trace is characterized by two maxima, whereas the angular velocity exhibits a single maximum, followed by a minimum during the toe-off phase.

In Figure 6c, both the *shuffling* and *trembling in place* phenotypes are zoomed. The *trembling in place* interval is approximately 81–83 s. As one can see, traces during FOG are quite different from the regular gait, although only in the *shuffling* intervals a sort of periodicity is still appreciable.

We then sought deeper insight into the typical features of the gyro and sEMG traces. For this reason, in Figure 6d, a single regular step is displayed. During the swing phase, there is a gyro maximum around *t* = 17.4 s where the sEMG has a local minimum related to the TA relaxation. Figure 6e,f depicts, respectively, the traces related to a single shuffled step and to step attempts during *trembling in place*. In particular, we notice that, in the shuffled step (Figure 6e), only one sEMG maximum is present (*t* = 76.45 s), while in *trembling in place* both traces are almost flat (Figure 6f).

Based on those considerations, we developed an algorithm to distinguish regular gait from FOG episodes in real-time.

**Figure 7:**
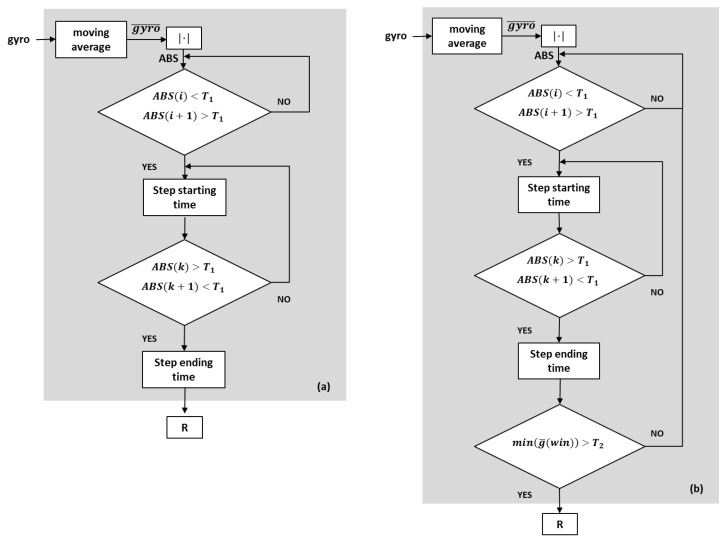
Flowchart of: (**a**) the FOG detection algorithm; and (**b**) the improved FOG detection algorithm.

The algorithm blocks are reported in the flow chart of Figure 7a. First, we smooth the gyro trace by calculating its moving average ($\bar{gyro}\left(i\right)$, with *i* = 1, *N* where *N* is the last acquired sample). Then, the normalized absolute value of the averaged angular velocity (ABS) is calculated:

(1) $$\mathrm{ABS}=|\bar{gyro}|$$

Now, a step time window is fixed. To this aim, we introduce a threshold T_1_, constant and independent of the studied subject (T_1_ = 0.01). For each value of the index *i*, ABS is compared with T_1_. When ABS passes from being lower to being higher than T_1_, the *starting* time of the step or step attempt is fixed.

Now, a new index k varying between *i* + 1 and *N* is introduced. When ABS returns lower than T_1_, the *ending* time of the step or step attempt is fixed. The time elapsed between starting time and ending time is now called step window (*win*). (In the case that the subject is voluntary resting, the gyro trace remains below T_1_ and *win* is not defined. This corresponds to the first “NO” branch).

Finally, the algorithm calculates the ratio (*R*) between the maximum value of ABS in the single step/step attempt and the corresponding sEMG value recorded:

(2) $$R=\frac{\max\left(\mathrm{ABS}\right)}{\mathrm{sEMG}{|}_{{t=t}_{\max\left(\mathrm{ABS}\right)}}}$$

Relying on the considerations above, we expected that the values of *R* in regular steps are much higher than in shuffled or attempted steps. We calculated *R* in all performed tests and compared it with the reference statements by the doctors. As a result, systematic errors occurred in regular gait, since in 50% of the classified steps the value of *R* was as low as during FOG.

**Figure 8:**
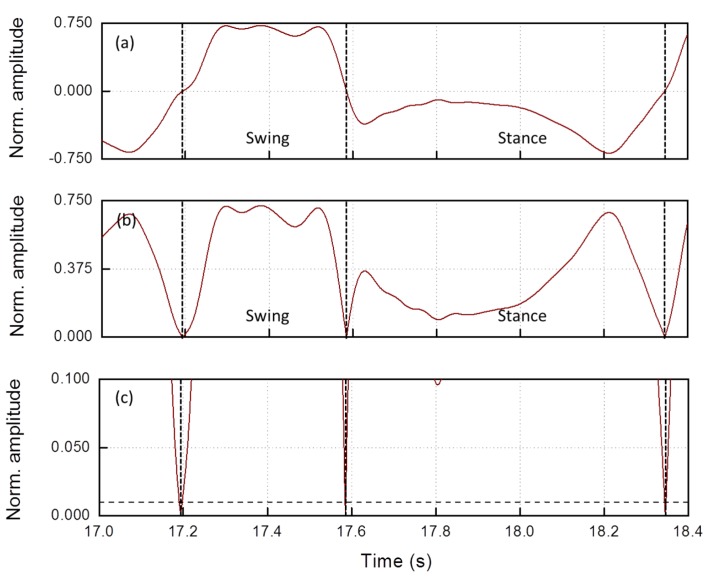
(**a**) An example of the normalized gyro trace of a single regular step recorded on the TA; (**b**) absolute value of the angular velocity; and (**c**) zoom around the threshold T_1_ = 0.01.

To comprehend the reason of this systematic error, we re-drew (Figure 8a) the gyro trace of a single regular step (this trace coincides with Figure 6d and is repeated here for the sake of clarity), calculated its absolute value (Figure 8b) and compared it with T_1_ on an expanded scale (Figure 8c). The *swing* and the *stance* phase are indicated in the figure. Passing from the swing to the stance phase and vice versa, the trace crossed T_1_ (arrows) and, consequently, the algorithm erroneously classified the two phases of the same step as two different *wins*(a swing *win* and a stance *win*). The error comes from the fact that, in the stance phase, in correspondence of the ABS maximum, the sEMG was quite active (toe-off), resulting in a low *R* value. Therefore, each regular step was erroneously classified by the algorithm as the sequence of a regular step (swing) and a FOG (stance).

To avoid this error, we had to disregard the stance win in regular steps. Actually, in stance phase, the gyro trace was always negative, but, in regular steps, it was more negative than in FOG (see Figure 6d–f). Thus, we introduced in the algorithm a negative threshold T_2_ for the angular velocity, so that, if the minimum value in the stance win is less negative than T_2_, the step is classified as irregular and only in this case the algorithm calculates *R* (T_2_ = −0.4 in all the tests performed on all the subjects). In Figure 7b, there is sketched the flow chart of the correct algorithm with this further block added: if min($\bar{g}\left(win\right)$) > T_2_ the algorithm calculates the new value of R.

R is now referred to as an index for FOG detection in real time.

### 3.2. Results

**Figure 9:**
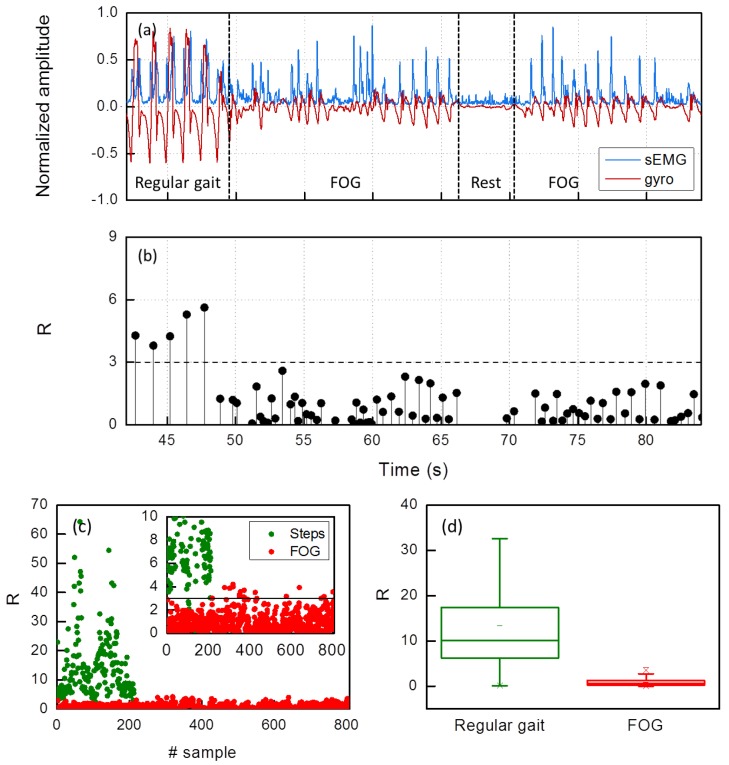
(**a**) An example of the sEMG (blue) and gyro (red) traces recorded on the TA (coinciding with a portion of Figure 6a, re-reported here for the sake of clarity); (**b**) the corresponding *R* values on the same time scale; (**c**) a scatter plot of all the tests; and (**d**) a box plot of all the tests.

The FOG index *R* was calculated in all the tests. Hereafter, we show that *R* assumes very different values when calculated in regular gait or in FOG. To this aim, in Figure 9a (which is portion of Figure 6a), we have selected the sequence of a few regular steps, a few shuffled steps, a rest interval (66–70 s), a few shuffled steps and a *trembling in place* FOG episode (81–83 s). The correspondent values of *R* are drawn in Figure 9b.

As one can see, we can define a demarcation level for *R* (*R* = 3), above which its values (black dots) correspond to regular steps and below which they correspond to FOG. It is worth noticing that the rest time interval is correctly recognized, so that *R* is not calculated at all in that interval (66–70 s). Figure 9b is a representation of an offline evaluation of FOG episodes occurrence. However, the algorithm displayed in Figure 7b runs continuously on the microcontroller embedded in the device and, as *win* is defined, *R* is calculated and compared with the demarcation level. Now, since, in the case of FOG, *win* is always in the order of 200 ms, we can likely affirm that the algorithm operates in real time.

In Figure 9c, the global scatter plot related to all the tests on all the subjects is depicted as function of the sample number. The total amount of points in the scatter plot is 1020, 808 of which are the sums of shuffled steps and step attempts during *trembling in place* (red dots) and 212 are regular steps (green dots).

The inset of Figure 9c is a zoom around the demarcation level *R* = 3. As a result, comparing our findings with the reference statements by the doctors, 98% of the shuffled or attempted steps have a value of the index *R* below 3, 95% of regular steps have a value above it, corresponding to 2% false negative and 5% false positive, respectively.

We wish to underline that these percentages refer to the detection of single shuffled/attempted steps inside FOG episodes, not only to the detection of the episodes themselves. In Figure 9d, the *R* box plot of the same data is shown to highlight the statistical distribution of FOG and regular steps.

## 4. FOG Phenotype Index

### 4.1. Methods

FOG subtypes may have different underlying pathophysiology [8]. The distinction between the *trembling in place* and the *shuffling forward* FOG phenotypes would allow to uncover possible different responses to treatments and to asses the associated risk of falls, in patients with PD.

In this section, we present an algorithm for distinguishing the *trembling in place* and the *shuffling forward* FOG based on the fusion of the sEMG and gyro traces in the frequency domain. In the *trembling in place* FOG, the trunk inclination consequent to an abrupt involuntary block of the gait increases the postural instability which obviously reflects in an increased fall probability [9]. Therefore, our system can be useful for definitely assessing the risk of fall and injuries related to one rather than to the other phenotype.

**Figure 10:**
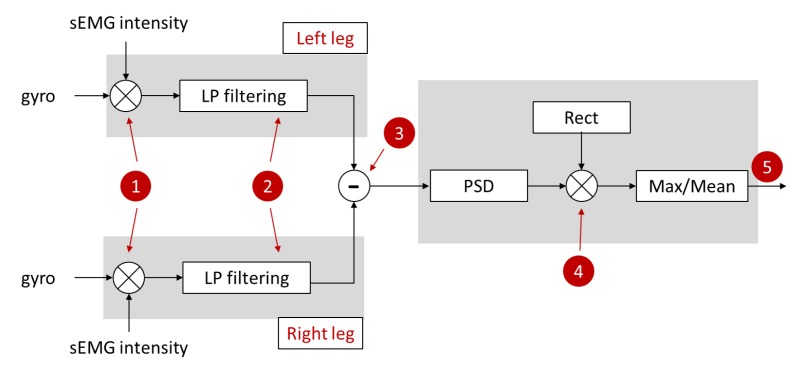
Sketch of the algorithm blocks implemented in the frequency domain for the offline distinction of the FOG phenotypes.

**Figure 11:**
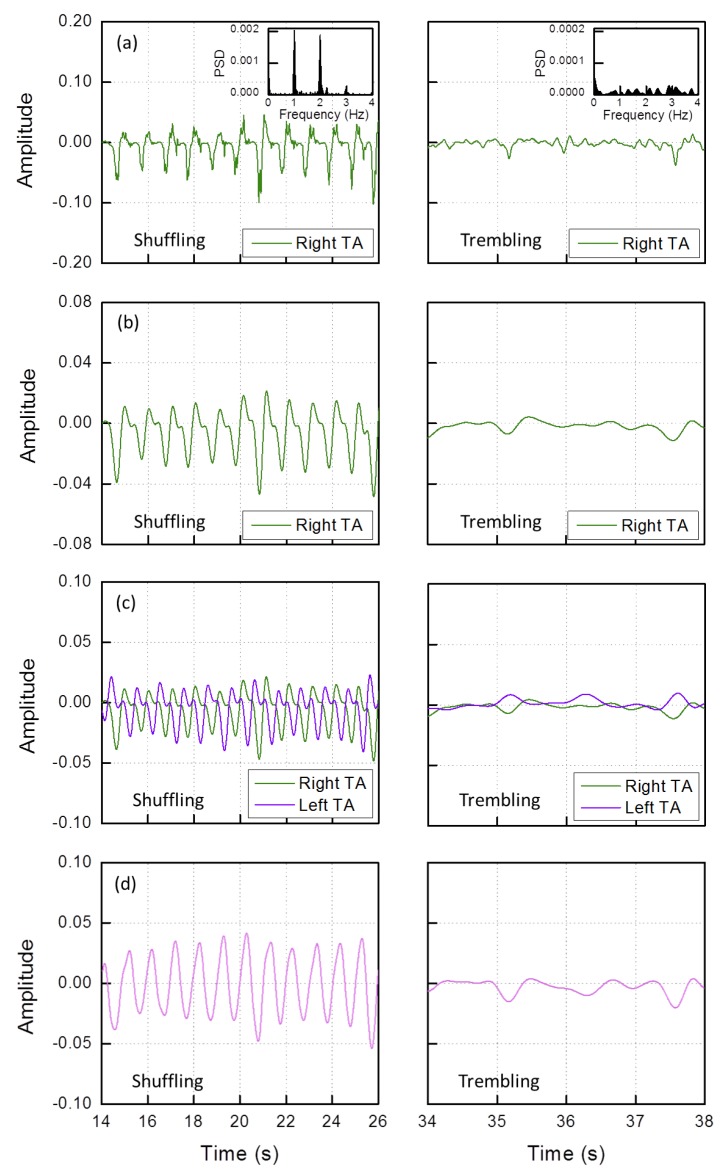
*Shuffling* (left) and *trembling* (right) FOG: (**a**) product of the normalized sEMG and gyro traces (inset: power spectral density); (**b**) low pass filtering at 2 Hz; (**c**) left and right leg traces; and (**d**) result of the subtraction of the right and left leg traces.

In this case, traces of the two legs need to be considered. A sketch of the algorithm blocks is reported in Figure 10. The first block (1) consists in calculating the product between the normalized sEMG and gyro traces during a FOG episode. The resulting product relative to a sample test is shown in Figure 11a for one leg experiencing first a *shuffling* and then a *trembling in place* FOG. The device was positioned on TA.

As expected, during the *shuffling* FOG episode, a series of negative peaks is present, since sEMG maxima correspond to gyro negative minima. On the contrary, during the *trembling in place* episode, the product varies randomly around zero.

The periodicity of the *shuffling* and the randomness of the *trembling in place*, FOG are more evident in the Power Spectral Density (PSD) displayed in the insets. The PSDs outline that the *shuffling* FOG peaks lie in the frequency range below 2 HZ (in the *trembling* case the scale is 0.1×).

The subsequent block (2) consists in a low pass filtering at 2 Hz by means of a Finite Impulse Response (FIR) filter. The result is shown in Figure 11b.

Now, we consider the traces of both the legs. Looking at Figure 11c, we see that the two legs traces are in antiphase in the *shuffling* interval while they are randomly varying in the *trembling in place* one. Thus, block (3) consists in subtracting the left from the right leg trace.

**Figure 12:**
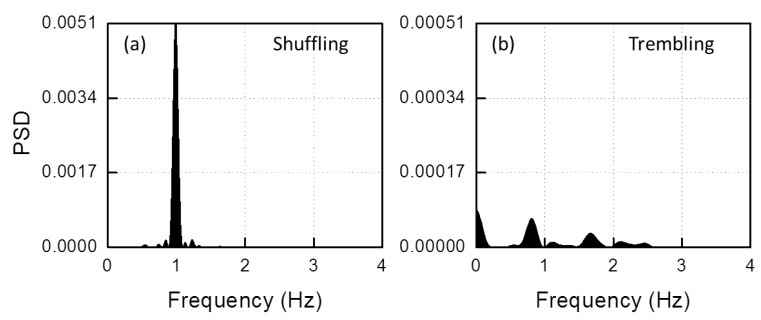
Power spectral density of the trace displayed in Figure 11d during: (**a**) *shuffling* FOG; and (**b**) *trembling* FOG.

The result is shown in Figure 11d. Subtraction gives a sinusoidal curve only in the *shuffling* interval and a value randomly varying around zero in the *trembling in place* interval. The PSDs displayed in Figure 12 outline these features (again, in the *trembling in place* case, the PSD amplitude scale is 0.1×).

Finally, in block (5) of Figure 10, the ratio between the maximum value of the PSD and its geometric mean in that frequency interval is calculated. We refer to this quantity as the FOG phenotype index (PI):

(3) $$\mathrm{PI}=\frac{\max\left(\mathrm{PSD}\right)}{{\mathrm{mean}}_{geom}\left(\mathrm{PSD}\right)}$$

### 4.2. Results

We calculated the value of PI for all the recordings on all the studied subjects. The average values of PI are reported in Table 1. As one can see, the two PI values calculated in the *shuffling* FOG and in the *trembling in place* FOG episodes are extremely different and therefore this index well characterizes the FOG phenotype. The quantity PI could be very useful in remote assistance of patients. In fact, during a long-time home monitoring, all the PI values are recorded in time. The prolonged recording of PI in a free living-like context would provide additional evidence about distinct pathophysiological mechanisms in FOG phenotypes and their associated risk of falls. In addition, such experimental approach would clarify possible differences in the response to treatments in patients with PD.

## 5. Analysis of Antagonist Muscles Activation Patterns during and Outside FOG

### 5.1. Methods

The pathophysiology of FOG is not well understood yet. Many hypotheses are presented, and they are not mutually exclusive. A better understanding of its mechanisms may lead to the development of effective therapeutic strategies. We hope that recording contemporarily the activity patterns of the antagonist leg muscles in free living-like conditions can add information in the current debate around the pathophysiology of FOG.

In this section, we study the *type* of activity, i.e., if the muscle is contracted or stretched. In this set of tests, two devices were positioned on the same leg: one recording the TA sEMG trace, the other recording the GC sEMG trace.

The conventional preprocessing of the raw sEMG signal recalled in Section 3 quantifies the *intensity* of the activity, but does not clarify the *type*. This information, on the contrary, can be derived studying the low frequency portion of the sEMG signal spectrum.

As mentioned, in general, this spectrum portion includes motion artifacts and hum noise, but using a wearable system they are intrinsically reduced since the device is battery powered and electrodes are not wired. Moreover, an accurate skin preparation helps reduce certain components of artifacts [56]. In a previous work, we already demonstrated that the muscle activity *type* in healthy subjects can be identified with a wearable system studying the sign of the LP filtered sEMG signal below 10 Hz [58]. In particular, the positive sign was related to contraction and the negative sign to stretching.

**Figure 13:**
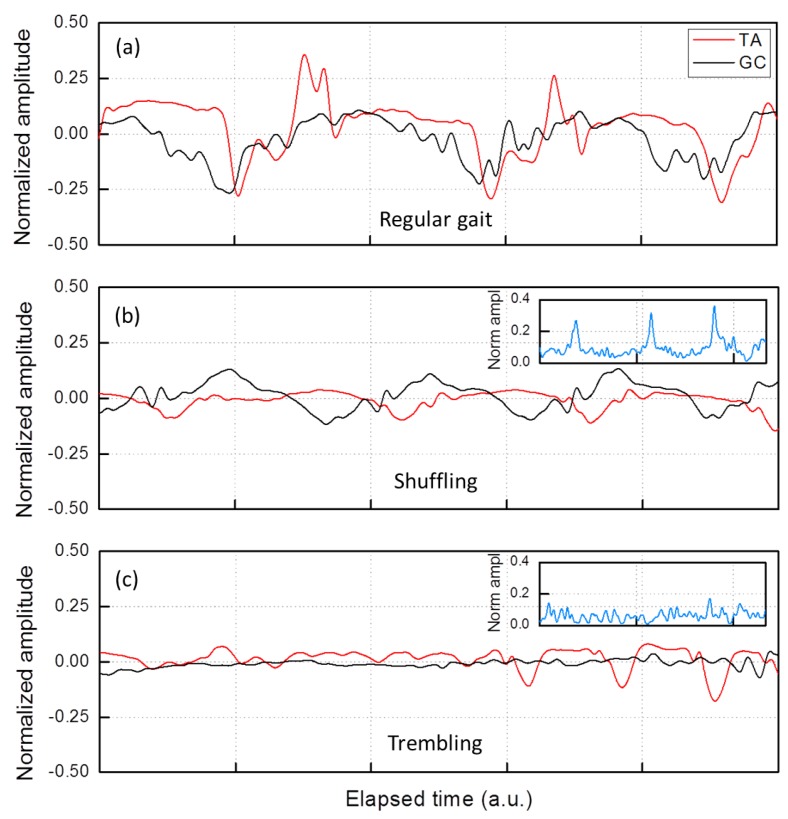
Muscle activity type traces relative to TA (red) and GC (black) of a single patient (P1): (**a**) outside FOG; (**b**) during a *shuffling* FOG; and (**c**) during a *trembling* FOG. The insets show the intensity in the same time interval. The timescale is the same in the three plots.

Now, we applied this unconventional processing to the lower limbs antagonist muscles of patients with PD during and outside FOG episodes. In Figure 13, the *type* traces relative to TA (red) and GC (black) of the same leg of a specific patient (P1) are displayed. The three plots of Figure 13 refer to three distinct gait situations during the same test. For convenience, the plots were time normalized and are reported on the same timescale.

### 5.2. Results

As an example, we report the pattern of muscles activation in a typical patient (patient P1) presenting both shuffling forward and trembling-like FOG episodes in Figure 13. As shown in Figure 13a, the *type* traces of both TA (the main dorsal flexor muscle of the foot) and GC (the main plantar flexor muscle of the foot) during regular gait exhibit a periodic pattern. In addition, during the *shuffling* FOG (Figure 13b), we find a periodic pattern in both the antagonist muscles. On the contrary, during the *trembling in place* FOG (Figure 13c), TA and GC have different features. Indeed, while the TA exhibits repeated contraction and stretching, the GC remains essentially stretched (being negative), not allowing to identify a standardized and recurrent pattern of muscles activation. To get deeper insight, we evaluated also the *intensity* of the GC stretching pattern in the *trembling in place* FOG episode of the same patient. This is reported in the inset of Figure 13c. For comparison, the *intensity* of the GC activity pattern during a *shuffling* FOG episode is drawn in the inset of Figure 13b. Complementing information on the activity *type* and *intensity*, the GC activity during the *trembling in place* FOG (Figure 13c) and, to a lesser degree, during *shuffling forward* FOG (Figure 13b) is characterized by a continuous stretching of very low entity.

**Figure 14:**
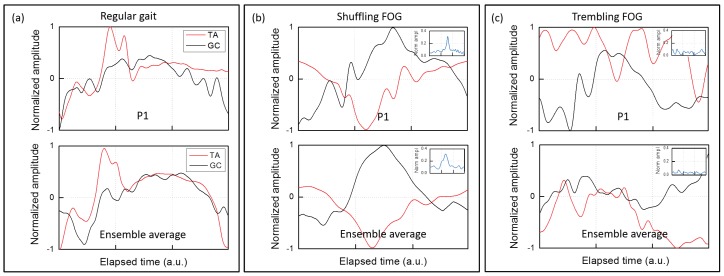
sEMG traces for the GC and TA recorded in the time interval Δt = 1 s referring to: (**a**) a single regular step of patient P1 (top plot) and the ensemble average of single regular steps (bottom plot); (**b**) a single shuffled step of patient P1 (top plot) and the ensemble average of single shuffled steps (bottom plot); and (**c**) a trembling episode of patient P1 (top plot) and the ensemble average of trembling episodes (bottom plot).

The ensemble averaging of steps during regular gait, shuffling forward and trembling-like FOG episodes with a Δt = 1 s time interval confirms these findings. In Figure 14a, we show traces referring to a single regular step of patient P1 (top plot) compared with the ensemble averaging of ten regular steps made by seven patients, OFF and ON therapy, (bottom plot). In Figure 14b we show traces referring to a single shuffled step of patient P1 (top plot) compared with the ensemble averaging of ten shuffled steps made by patients presenting shuffling forward FOG episodes (three subjects), OFF and ON therapy (bottom plot). As a result, one can easily recognize the same trace features for both GC and TA in patient P1 and in the ensemble average during regular gait and shuffling forward FOG episodes. Otherwise, in Figure 14c, we confirm the lack of a standardized and periodic pattern of muscles activation during trembling-like episodes also analyzing ensemble averaging. Finally, when considering the activity intensity of leg muscles during FOG episodes in the ensemble averaging, we confirm that the GC presents a defective activity both during the trembling in place FOG (inset of Figure 14c) and, to a lesser degree, during shuffling forward FOG (inset of Figure 14b). We therefore hypothesize that during FOG the foot is shuffled forward or is completely stopped with a trembling-like appearance owing to a variable deficit in the “toe-off” phase. “Toe-off” is a predictable event during regular gait cycle (Figure 5), involving the strong activation of the ankle flexor muscles (i.e., GC) for the plantar-flexion of the foot [59]. Physiologically, proprioceptive signals from extensor muscles (i.e., TA) contribute to the transition phase from stance to swing of the leg, by modulating the activity of ankle flexor muscles in relation to changes of leg loading [60,61]. Indeed, when a leg is under load, ankle flexor muscles are inhibited by the activity of ankle extensor proprioceptors [60]. Previous experimental studies [62,63,64] already demonstrate abnormal anticipatory postural adjustments (APAs) in PD patients with FOG, causing defective shift of the body weight during step execution. We therefore speculate that the defective unloading of the leg during the gait cycle, due to abnormal APAs, is responsible for reduced activation of GC and consequently for the “toe-off” deficit, observed during FOG episodes. Based on these considerations, we conjecture that FOG is associated with abnormal patterns of activity in the TA and GC muscles leading to a prominent deficit in the “toe-off” phase of the gait cycle. According to previous findings [65], proprioceptive manipulation of legs muscles could help to overcome FOG episodes by strengthen APAs and GC activation during the transition from the stance to the swing phase of the step.

To our knowledge, this is the first time that the activity type of the lower limb antagonist muscles was analyzed during and outside FOG episodes in free living-like condition.

## 6. Conclusions

In this paper, we propose a wearable sensing system for automatic, continuous and ubiquitous monitoring of FOG in patients with PD. The novelty of our study lies in the fact that the present device merges information from gyroscopes with sEMG, monitoring contemporarily legs rotational motion and muscle potential patterns. The use of both inertial and electrical signals allowed obtaining an accurate and FOG detection, with only 2% false negative and 5% false positive in the automatic assessment of seven patients presenting 99 FOG episodes during 57 min of recording in free living-like environment.

The good performance of the proposed sensing system would allow real-time FOG detection for prevention strategies of falls and injuries in PD patients with FOG. Several recent studies have demonstrated the efficacy of cueing devices in overcoming FOG episodes in PD patients [66,67,68]. Accordingly, early and accurate FOG detection would promptly trigger specific stimuli able to improve FOG management or prevent injuries by using specific protective devices such as airbags [69].

A highly innovative aspect of the work is that, in accordance with previous reports about different underlying pathophysiology in FOG subtypes [10], we developed an algorithm using a frequency-domain processing of the signal fusion for the differentiation between the *trembling in place* and the *shuffling forward* FOG phenotypes. This algorithm can potentially help to highlight differences between the *trembling in place* and the *shuffling forward* FOG in response to treatments and in the associated risk of falls, thus possibly uncovering distinct pathophysiological underpinnings in PD patients with FOG.

Another potentiality of the present device lies in the fact that it can detect the *type* (contraction or stretching) and the *intensity* of leg antagonist muscles activity performing sEMG in free living-like environment. This analysis performed during and outside FOG episodes allows hypothesizing a biomechanical and pathophysiological definition of FOG. To our knowledge, this is the first time that the activity *type* and *intensity* of the lower limb antagonist muscles were analyzed simultaneously during and outside FOG episode in free living-like conditions. This opens up alternative therapeutic approaches, such as proprioceptive manipulation, to support the transition from the stance to the swing phase of the step in patients with PD and FOG.

Finally, the continuous acquisition and storage of the patient physiological parameters (number of FOG episodes, FOG phenotype and muscle activity type) permits the development of an up-to-date electronic agenda, specific for each patient, thus opening to telemedicine applications, including tele-rehabilitation. Several studies [70,71,72,73] have already shown practical perspectives of wearable sensors use in the home environment to remotely monitor patients’ clinical state and improve therapeutic strategies. The objective and long-term measures by means of wearable sensors in free living-like conditions would support patients’ physical activity by giving feedback about motor performance and tailored instructions [74], such as lengthen the stride length during walking to limit the sequence effect (step-to-step decrease in amplitude) and thus potentially reduce FOG occurrence [75].

## Figures and Tables

**Table 1:** PI values averaged on 99 FOG episodes.

FOG Type	PI
Shuffling	109.34 ± 2.14
Trembling	3.18 ± 1.29

## Abbreviations

The following abbreviations are used in this manuscript:

FOG	Freezing of Gait
PD	Parkinson’s Disease
EMG	Electromyography
GC	gastrocnemius
TA	Tibialis Anterior
IMU	Inertial Measurement Unit
sEMG	Surface EMG
RMS	root mean square
CMRR	common mode rejection ratio
SNR	signal to noise ratio
HP	high-pass
LP	low-pass
ABS	normalized absolute value of the averaged angular velocity
R	ratio between the maximum value of ABS and the corresponding sEMG value
PSD	Power Spectral Density
FIR	Finite Impulse Response
PI	FOG phenotype index
APA	anticipatory postural adjustments
MDS-UPDRS	Modified Disorder Society-Unified Parkinson’s Disease Rating Scale
